# American Rhinological Association

**Published:** 1888-10

**Authors:** 


### AMERICAN RHINOLOGICAL ASSOCIATION.

### First Day—Morning Session.

The sixth annual meeting was held at Cincinnati, Ohio, September 12, 13, and 14.

The Association was called to order by the President, at ten o'clock.

Dr. A. B. Thrasher, of Cincinnati, delivered an address of welcome, after which the President delivered the annual address, in which he recalled the progress that has been made in rhinology.

Dr. J. E. Schadle, of St. Paul, Minnesota, read a report of "A Case of Chorea of the Soft Palate, caused by the Hypertrophy and Hyperæsthesia of the Mucous Membrane Covering both Inferior Turbinated Bodies."

He said some forms of headache and of spasmodic asthma are, in many instances, the outgrowth of a nasal polypus, a turbinated thickening, or a septal deformity. These reflex neurotic disturbances disappear after appropriate measures of treatment have been directed towards the removal of their cause.

A patient was examined by Dr. Shadle on April 16, 1888. She suffered from constipation and flatulency quite frequently. The heart's action was more or less excited; the heart beat tumultuously when ascending a flight of stairs, or some other elevation. She complained of marked obstructed nose-breathing; her voice was impaired, especially when singing. The principal difficulty for which she was sent to him was the spasmodic movement of the velum palati.

Faucial examination revealed distinct rythmical choreiform movements of the velum palati, accompanied by a peculiar clicking sound, distinctly audible for a distance of twelve or fifteen feet from the patient. Anterior rhinoscopic examination revealed chronic hypertrophy of the inferior turbinated bones, more especially the middle one, whose redundant tissue caused pressure on the septum and produced obstruction of the middle meatus.

Treatment was mainly surgical. Snaring was impracticable. By the use of Cohen's post-nasal cutting forceps and the electro-cautery, he reduced the growth entirely, the procedure being followed by cessation of the choreiform movements.

### Afternoon Session.

Dr. Thos. F. Rumbold, of St. Louis, Missouri, read a paper on "The Etiology and Pathology of Nasal Diseases." The etiology of a disease is of the greatest import. A perfect understanding of its causes and its mechanism is the goal of the pathologist. As regards heredity in the causation of rhinitis, he had long been convinced that such a theory was erroneous. The histological facts concerning cell-life would prove a successful contravention of the theory of heredity.

### Second Day—Morning Session.

A paper entitled "The Relation of Nasal Diseases to Other Affections, Including the Brain and Nervous System," was read by Dr. John North, of Keokuk, Iowa.

There is no organ, he said, in the entire body that has been so neglected as the nose. Obscure and obstinate diseases have not yielded to treatment because it has been directed to the effect of nasal reflex troubles and not to the nose, the cause of the trouble.

He claimed that in 1886 he had shown almost conclusively that certain cases of neurasthenia were the result of chronic naso-pharyngeal catarrh, and reported several cases to substantiate his argument, and which yielded to treatment applied to the naso-pharynx.

Dr. Thos. F. Rumbold read a paper on "The Effect of Nasal Inflammation on the Mind." A normal condition of the mind depends upon a normal supply of blood to the normal brain. If an acute inflammation in the nasal passages is accompanied by mental manifestations, mental incapacity of a more severe character must accompany a chonic rhinitis.

A paper was presented on "The Etiology and Pathology of Acute Catarrh," by Dr. J. G. CARPENTER, of Stanford, Kentucky.

Among the most prominent causes, he mentioned occupations attended with dust, smoke, excessive moisture, or dry, dusty atmosphere, going about insufficiently clad, the evil results of ordinary slippers, etc.

Pathologically, acute catarrh is divided into three stages: (1) The dry stage, attended with hyperæmia, redness, heat, tumefaction, and pain of mucous membrane, the latter presenting a red, dry, or dark-red appearance. (2) Moist stage, supplementing the first; there is transudation of serum, diapedesis of the white corpuscles into the connective tissue, cell-proliferation, and organization of lymph. (3) The presence of pathological conditions, with denudation of the epithelium and the presence of a raw surface, and the excessive mucous secretion is changed to a muco-purulent or purulent one.

AFTERNOON SESSION.

Dr. R. S. KNODE, of Fort Wayne, Indiana, read a paper on "Intra-Nasal Obstruction."

He said, of all the mucous surfaces that of the nose is perhaps the most sensitive to irritating gases, vapors, and dust of different kinds, as well as sudden changes of atmosphere. Intra-nasal obstructions may be temporary or permanent, partial or complete, owing to the extent of inflammation or injury that produces them. Temporary occlusion is usually the result of some one of the forms of acute rhinitis. From severe attacks, or from the effects of repeated colds, the membrane may be found overwhelmed with plastic material, with a loss of nerve power, a pale, flabby, wrinkled condition, somewhat resembling polypus, but more sensitive to the touch, and completely filling up both cavities of the nose. In this condition he had found nothing to act as promptly as to first use a solution of cocaine, applied with a probe and cotton, and followed with mildly stimulating solutions of chromic acid, ten grains to one ounce, after which the vaseline treatment should be used. If stenosis should return, a second application of the acid may be made, or slippery-elm plugs, slightly moistened with a weak preparation of cocaine and listerine.

Dr. E. G. KEGLEY, of Cedar Rapids, Iowa, reported "A Case of Enlarged Tonsils, with Peculiar Symptoms, Relieved by the Galvano-Cautery Snare."

The patient was 43 years of age male; first consulted him in January, 1888, with regard to a trouble which the patient termed "drooling." He presented the appearance of one suffering from hay-fever. His eyes were red and swollen, with excessive lachrymation, constant flowing and wiping of the nose, and spitting of clear fluid. Examination revealed the nasal mucous membrane highly congested, and completely closing the nasal passages. The patient's mouth was constantly filled with saliva, and tonsils enormously enlarged, filling the back part of the mouth and throat. Dr. Kegley cocainized the tonsils and removed them both at one sitting by means of the galvano-cautery snare, the patient having experienced no pain whatever. He saw the patient the next day, who said the trouble had ceased almost simultaneously with the removal of the tonsils. He has had no further trouble.

THIRD DAY—MORNING SESSION.

Dr. A. G. HOBBS, of Atlanta, Georgia, read a paper on "The Surgery of Gummatous Growths of the Nasal Cavities," and reported four cases. In the first case he cocainized the nasal passages with a 5 per centum spray, then attempted to introduce a Blake's snare, but found it impossible to engage the tumor in the loop. He therefore introduced a cutting spoon, and several large masses of the growth were cut away. Four more sittings, at intervals of three days, enabled him to remove the growth entirely. The patient has completely regained her health.

AFTERNOON SESSION.

Dr. A. B. THRASHER, of Cincinnati, read a paper on "Surgical Treatment of Nasal Catarrh," in which he said that there are forms of nasal catarrh that can only be benefited, and not cured, by medicinal treatment. When we have mechanical obstructions to the lumen of the naris of a hypertrophic, hyperplastic, or neoplastic character, surgery offers the most direct path for the removal of the difficulty. What is the best method of procedure? There are cases in which the application of a chemical caustic answers the purpose well. Woakes' gouge or plow will do the work, and by the aid of cocaine will do it rapidly and painlessly.

In uncomplicated cases of chronic hypertrophic rhinitis, the conditions to be met are: (*a*) Obstructions to lumen of the naris, and (*b*) abnormal condition of mucous glands. The causal indications would at once suggest removal of the redundant tissue. The means employed should look to the accomplishment of this end, and with the least resulting scar-surface, so that the natural condition of the epithelial covering might be as nearly as possible simulated. To reach this result he had met with the most marked success in the use of the galvano-cautery knife. He first anæsthetizes the part with cocaine, then introduces the knife to the posterior part of the hypertrophy, turns the sharp edge toward the tissue to be cut, heats the knife white hot, and cuts deeply, drawing the knife forward. The result is a deep linear scar in an antero-posterior direction through the hypertrophied tissue. He cuts deeply enough to destroy a portion of the submucous erectile tissue.

After electing officers and selecting Chigago as the next place of meeting, in September, 1889, the Association adjourned.

